# Mechanism of Dyslipidemia in Obesity—Unique Regulation of Ileal Villus Cell Brush Border Membrane Sodium–Bile Acid Cotransport

**DOI:** 10.3390/cells8101197

**Published:** 2019-10-03

**Authors:** Shanmuga Sundaram, Balasubramanian Palaniappan, Niraj Nepal, Shaun Chaffins, Uma Sundaram, Subha Arthur

**Affiliations:** Department of Clinical and Translational Sciences, Appalachian Center for Cellular transport in Obesity Related Disorders, Joan C. Edwards School of Medicine, Marshall University, 1600 Medical Center Drive, Huntington, WV 25701, USAsundaramu@marshall.edu (U.S.)

**Keywords:** obesity, ASBT, Na–bile acid cotransport, Zucker rats, TALLYHO mice, Farnesoid X receptor, bile-acid-associated protein

## Abstract

In obesity, increased absorption of dietary fat contributes to altered lipid homeostasis. In turn, dyslipidemia of obesity leads to many of the complications of obesity. Bile acids are necessary for the absorption of dietary fat. In the mammalian intestine, apical sodium-dependent bile acid cotransporter (ASBT; SLC10A2) is exclusively responsible for the reabsorption of bile acids in the terminal ileum. In rat and mice models of obesity and importantly in obese humans, ASBT was increased in ileal villus cells. The mechanism of stimulation of ASBT was secondary to an increase in ASBT expression in villus cell brush border membrane. The stimulation of ASBT was not secondary to the altered Na-extruding capacity of villus cells during obesity. Further, increased Farnesoid X receptor (FXR) expression in villus cells during obesity likely mediated the increase in ASBT. Moreover, enhanced FXR expression increased the expression of bile-acid-associated proteins (IBABP and OSTα) that are responsible for handling bile acids absorbed via ASBT in villus cells during obesity. Thus, this study demonstrated that in an epidemic condition, obesity, the dyslipidemia that leads to many of the complications of the condition, may, at least in part, be due to deregulation of intestinal bile acid absorption.

## 1. Introduction

Obesity is an epidemic in the United States with nearly two out of five adults reported to be obese (https://www.cdc.gov/obesity/data/adult.html). Dyslipidemia is an inevitable complication of obesity, which in turn is central to many of the complications of obesity including metabolic syndrome, nonalcoholic steatohepatitis, nonalcoholic fatty liver disease, coronary artery disease, cerebrovascular disease, pancreatitis, and so forth. [1,2,3,4]. Intestinal absorption of lipids is dependent on the availability of bile acids. Thus, an important feature of altered lipid homeostasis in obesity, increased absorption of dietary fat, is thought to be secondary to increased bile acid levels in the intestine [5,6,7]. Thus, altered bile acid homeostasis likely contributes to the dyslipidemia of obesity. 

Bile acids are amphipathic molecules that are produced as end products of cholesterol catabolism in the liver and secreted in the proximal small intestine to facilitate digestion and absorption of lipids. The bile acids are later reabsorbed in the distal small intestine and enter enterohepatic circulation [8]. Enterohepatic circulation of bile acids plays a major role in the maintenance of bile acid homeostasis and is highly regulated by hepatic and intestinal factors [9,10,11,12,13]. In conditions such as inflammatory bowel disease (IBD), malabsorption of bile acids disrupts its homeostasis and results in malabsorption of lipid and lipid soluble vitamins, increased colonic permeability, and steatorrhea and diarrhea [14,15,16,17]. In contrast, during obesity, bile acid levels have been shown to be significantly increased in the intestine, thereby perhaps causing the enhanced absorption of dietary lipids [6,7]. Enhanced intestinal bile acids may be due to overproduction of bile acids by the liver, heretofore unreported, or secondary to increased absorption of bile acids by the enterocytes.

The sodium-dependent bile acid cotransporter (ASBT, SC10A2) exclusively enables reabsorption of bile acids in the distal ileum. It is localized to the brush border membrane (BBM) of absorptive villi but not crypt cells in the mammalian ileum. The favorable trans membrane Na-gradient required for ASBT is provided by basolateral membrane (BLM) Na-K-ATPase of villus cells [18,19]. Thus, altered bile acid homeostasis of obesity may be at the level of ASBT on the BBM of villus cells and/or altered Na-extruding capacity of the cells.

Additionally, ASBT has shown to be regulated by various nuclear factors such as farnesoid X receptor (FXR), hepatocyte nuclear factor 1α (HNF-1α), activator protein-1 (AP-1), and transcription factor Gata4 [20,21,22,23,24,25]. Increased ASBT promoter activity [24,25] has been shown to increase the expression of ileal ASBT in rats with streptozocin-induced diabetes mellitus. In contrast, as previously noted, ASBT activity and expression have shown to be decreased by inflammatory mediators in the chronically inflamed intestine leading to bile acid malabsorption [15,16,17]. Of all the transcription factors that have been implicated in the transcriptional regulation of ASBT, FXR appears to be most important [26]. This bile-acid-activated nuclear receptor protein is expressed in high levels in the intestine. It is known to regulate bile acid synthesis in the liver by repressing the synthesis of the enzyme CYP7A1, which is important for bile acid synthesis [8,27]. FXR has also been implicated in the regulation of other transporters such as Na:H exchange (NHE3) in colon cancer cells [28]. 

Despite the importance of ASBT in the maintenance of bile acid homeostasis and thus in turn lipid steady state, the altered regulation of ASBT which may lead to dyslipidemia of obesity is unknown. Thus, the aim of this study was to determine the mechanism of alteration of ASBT during obesity to better understand the etiology of dyslipidemia of obesity.

## 2. Materials and Methods

### 2.1. Animals

Genetically obese (*Lepr^fa^*) Zucker rats (OZR), males at 18 weeks of age, were used as one of the in vivo models of obesity to determine ASBT regulation in obesity. Leptin resistance as shown by OZR is strongly associated with extreme obesity and is a heritable trait [29]. They are characterized by hypertriglyceridemia, hypercholesterolemia, and metabolic syndrome [30,31]. Lean Zucker rats (LZR) were used as controls against OZR. Terminal ileum from the rats was used in all studies. Zucker rats were obtained from Charles River Laboratories International, Inc. 

TALLYHO mice (males), a polygenic model of obesity, were used at 18 weeks of age to study the differential regulation of ASBT in the terminal ileum. TALLYHO mice have been shown to develop moderate levels of obesity characterized by type 2 diabetes, hyperlipidemia, and hyperglycemia starting at eight weeks of age when fed with regular chow [32,33,34]. C57BL/6 mice have been widely used as controls for the TALLYHO mouse model in obesity research and thus 18-week-old C57BL/6 were used as controls for TALLYHO mice and all mice were obtained from The Jackson Laboratories.

The animals were included in the study after a week of acclimatization in the animal facility, and were maintained in 12 h/12 h light and dark cycle with unrestricted access to food and water. The animal studies were carried out strictly in accordance with the procedural and ethical regulations of Marshall University’s Institutional Animal Care and Use Committee.

### 2.2. Human Small Intestinal Samples

Human distal ileal mucosal samples were obtained during colonoscopy from patients without any known intestinal pathology according to a Marshall University IRB-approved and regulated protocol (IRB# 964144).

### 2.3. Villus Cell Isolation and Uptake Studies in Isolated Ileal Villus Cells

Villus cells were isolated from the ileum of the experimental animals by a calcium chelation technique as previously described [19] with few modifications. Briefly, isolated distal ileal villus cells were washed twice in TMA-HEPES buffer (50 mM KCl, 0.1 mM MgSO_4_, 50 mM Mannitol, 50 mM HEPES-Tris (pH 7.5), and 100 mM TMA-Cl) and suspended in 100 µL of the same buffer. Next, 10 μL of the cells from the suspension were added to 100 μL of reaction media containing 0.1 mM taurocholate (TCA; Sigma-Aldrich Corporation, St. Louis, MO, USA) with 100 μM ^3^H-taurocholate (PerkinElmer, Inc., Waltham, MA, USA), 50 mM KCl, 0.1 mM MgSO_4_, 50mM Mannitol, 50 mM HEPES-Tris (pH 7.5), and either 100 mM TMA-Cl or 100 mM NaCl. The reaction was stopped at two minutes by adding 1 mL of ice-cold TMA-HEPES buffer. The stopped reaction mixture was filtered on 0.65 μm Millipore (HAWP) filter and washed twice with ice-cold stop solution. The reactions were carried out in triplicate for each of the two reaction mixtures. The filter was dissolved in 5 mL of scintillation fluid (Ecoscint A, National Diagnostics), and radioactivity was determined in a Beckman 6500 scintillation counter (Beckman Coulter Inc., Brea, CA, USA).

### 2.4. In Vitro Cell Model

Rat small intestinal epithelial cells (IEC-18; ATCC CRL-1589, American Type Culture Collection, Manassas, VA, USA) at four days post confluence, were used as the in vitro model. These cells were maintained in Dulbecco’s modified Eagle’s medium (DMEM, Invitrogen–ThermoFisher Scientific, Waltham, MA, USA) supplemented with 10% (*v*/*v*) fetal bovine serum, 100 U/L human insulin, 0.25 mM β-hydroxybutyric acid, and 100 U/mL of penicillin and streptomycin, and were cultured in a humidified atmosphere of 10% CO_2_ at 37 °C. These cells were treated with the FXR agonist GW4064 (Sigma-Aldrich Corporation, St. Louis, MO, USA) [35,36] at a concentration of 500 nM/24 h.

### 2.5. Na-K-ATPase Assay

Na-K-ATPase activity was determined on isolated villus cell homogenates as previously described [37]. Na-K-ATPase activity was measured as inorganic phosphate release, which was expressed as nanomoles per mg of cellular protein per minute.

### 2.6. BBM Vesicle (BBMV) Preparation and Uptake Studies

BBMV from isolated villus cells were prepared by a method using Mg^++^ precipitation and differential centrifugation as previously reported [19,38]. Prepared BBMV were suspended and incubated in the appropriate reaction medium (100 mM choline chloride, 0.1 mM MgSO_4_, 50 mM HEPES-Tris (pH 7.5), 50 mM mannitol, and 50 mM KCl) and used for uptake experiments. BBMV uptake was performed by a rapid filtration technique. Briefly, 5 μL of BBMV was incubated in 95 μL reaction medium that contained 50 mM HEPES-Tris buffer (pH 7.5), 0.1 mM taurocholate with 100 μM ^3^H-taurocholate, 0.1 mM MgSO_4,_ 50 mM KCl, 50 mM mannitol, and 100 mM of either NaCl or choline chloride. Uptake was arrested after one minute by mixing with ice-cold choline chloride stop solution. The mixture was filtered through a 0.45 μm MCE membrane filter (MilliporeSigma, Burlington, MA, USA) and the filter was washed twice with 4 mL of ice-cold stop solution. The filter was then dissolved in 5 mL of scintillation fluid Ecoscint A (National Diagnostics, Atlanta, GA, USA) and radioactivity was determined.

For the BBMV kinetic studies, uptake experiments were performed at 15 s at varying extravesicular sodium taurocholate concentrations (10 µM, 25 µM, 50 µM, 125 µM, and 250 µM). Data that were derived from these experiments were analyzed with GraphPad Prism 7 (GraphPad Software Inc., San Diego, CA, USA) for Michaelis–Menten kinetics using a nonlinear regression data analysis to derive kinetic parameters *V_max_* and *K_m_*.

### 2.7. Western Blot

Western blot studies were performed using standard protocols, as previously described [39]. Brush border membrane protein extracts were used to determine ASBT protein expression in the experimental models of obesity. To determine ASBT protein expression in human samples, protein extracts were prepared from intestinal biopsy samples. For this study, an ASBT antibody reactive to rat, mouse, and human samples was used (bs-23146R, Bioss Antibodies Inc., Woburn, MA, USA) to detect ASBT protein expression. The following antibodies were used to detect the expression of the specific proteins: Farnesoid X receptor: FXR, sc-25309, Santa Cruz Biotechnology, Inc., Dallas, TX, USA [40,41]; ileal bile-acid-binding protein: ILBABP, AP26370PU-N, OriGene Global, Rockville, MD, USA [42]; organic solute transporter alpha: OSTα, SAB1306154, Sigma-Aldrich Corporation, St. Louis, MO, USA; and Ezrin: MA5-13862, Invitrogen Life Technologies, Carlsbad, CA, USA [43,44]. All the HRP-conjugated secondary antibodies were obtained from Abcam (Abcam PLC, Cambridge, MA, USA).

### 2.8. Protein Assay

To derive data in each of the individual uptake experiments, protein levels in cellular homogenates and BBMVs were determined using Bio-Rad DC^TM^ Protein Assay Reagents (Hercules, CA, USA) using the manufacturer’s instructions. For protein extracts used for Western blot experiments, protein levels were determined directly on Nanodrop 2000C Spectrophotometer (Thermo Fisher Scientific Inc., Waltham, MA, USA).

### 2.9. Statistical Analysis

All the results in this study are presented as means ± SE of ‘n’ number of experiments performed. Each of the ‘n’ number of uptakes was done in triplicate and represents cells obtained from different animals. Student’s t-test was performed for statistical analysis and the data was considered significant if the *p*-value was <0.05.

## 3. Results

### 3.1. Effect of Obesity on Villus Na–Bile Acid Cotransport in Zucker Rat Model of Obesity

**Figure 1:**
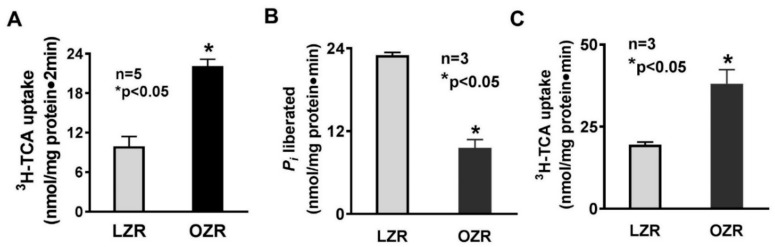
Na–bile acid cotransport and Na-K-ATPase in Zucker rat villus cells. (**A**) Na-dependent bile acid (^3^H-taurocholate or TCA) cotransport was significantly increased in intact villus cells from obese Zucker rats (OZR) compared to lean Zucker rats (LZR). (**B**) Na-K-ATPase activity was significantly reduced in villus cell homogenates from OZR. (**C**) Villus cell BBM Na–bile acid cotransport was also significantly increased in OZR.

Na-taurocholate uptake was significantly increased in isolated intact ileal villus cells from OZR compared to villus cells from LZR (Figure 1A: 9.9 ± 1.5 nmol/mg pro/ 2 min in LZR and 22.1 ± 1.05 in OZR; *n* = 5, *p* < 0.05). Na-K-ATPase in the BLM provides the favorable transcellular Na gradient for ASBT’s optimal activity, so Na-K-ATPase assay was performed in villus cell homogenates. Interestingly, Na-K-ATPase activity was found to be significantly decreased in OZR compared to LZR (Figure 1B: 23 ± 0.4 nmol/mg pro/min in LZR and 9.6 ± 1.2 in OZR; *n* = 3, *p* < 0.05). To determine if increased ASBT activity is also at the level of the cotransporter in the BBM, where the cotransporter ASBT is functionally active, BBMV uptakes were done. Na-dependent bile acid cotransport was significantly increased in villus BBMV from OZR (Figure 1C: 19.5 ± 0.8 nmol/mg pro/min in LZR and 38.1 ± 4.3 in OZR; *n* = 3, *p* < 0.05). These data demonstrated that the alteration of Na–bile acid cotransport in obesity is at the level of BBM ASBT in the villus cells, and not secondary to altered BLM Na-K-ATPase activity.

### 3.2. Effect of Obesity on the Kinetic Parameters of BBM Na–Bile Acid Cotransport

**Figure 2:**
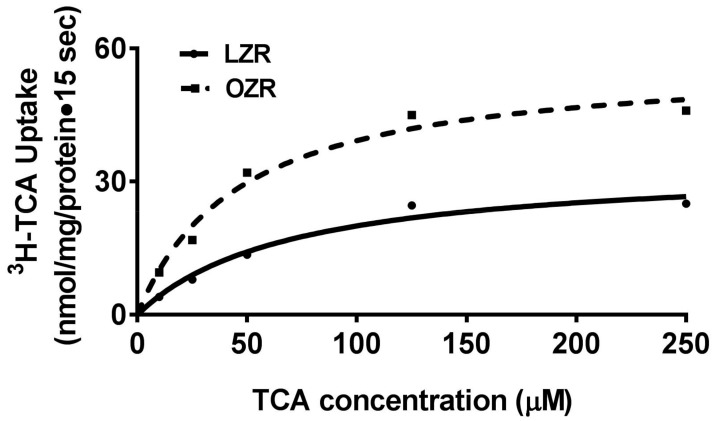
Villus cell BBM Na–bile acid cotransport kinetics in Zucker rats. A representative graph of kinetics of Na-taurocholate cotransport in BBMV prepared from OZR compared to LZR is shown. As the concentration of extravesicular taurocholate (TCA) increased, Na–bile acid uptake was stimulated, but subsequently became saturated in both the experimental conditions. The kinetic parameters derived from *n* = 4 of such experiments are shown in Table 1. While the maximal rate of uptake (*V_max_*) was increased in OZR, the affinity for taurocholate uptake (1/*K_m_*) was unchanged between OZR and LZR.

To determine the mechanism of stimulation of Na–bile acid cotransport in obesity, kinetic studies were performed. In both the experimental conditions, as the concentration of extracellular taurocholate was increased, the uptake of Na-dependent taurocholate was also stimulated and subsequently became saturated in all conditions (Figure 2). Table 1 shows the kinetic parameters derived from the kinetic experiments. As shown in the table, the maximal rate of uptake was found to be significantly increased in the villus BBMV from OZR compared to that from LZR. However, the affinity for bile acid uptake remained unchanged between the two experimental conditions. These results indicated that the increased villus Na-dependent bile acid cotransport in obesity is secondary to increased BBM cotransporter numbers.

### 3.3. Obesity Mediated Alterations in Villus ASBT Expression

**Figure 3:**
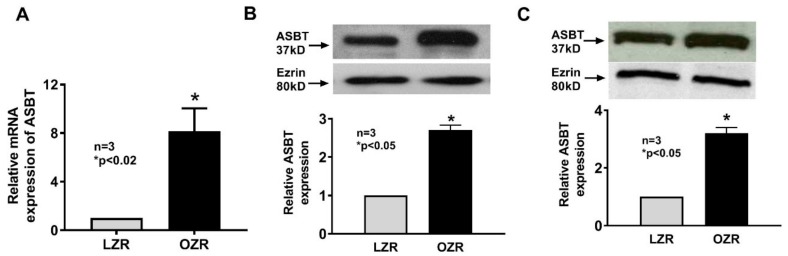
Effect of obesity on ASBT expression in Zucker rat villus cells. (**A**) ASBT mRNA expression was significantly increased in distal ileal villus cells from OZR compared to LZR. (**B**). ASBT protein levels were significantly increased in whole villus cell protein extract from OZR compared to LZR. (**C**) ASBT protein expression was also increased threefold in villus cell BBM from OZR compared to LZR.

To determine whether increased ASBT numbers were transcriptional, ASBT mRNA levels were measured and found to be increased in villus cells from OZR compared to LZR by real-time PCR (Figure 3A). Since mRNA does not necessarily correlate with protein, ASBT protein (37 kD) expression was determined in villus cells and found to be increased threefold in whole villus cell lysates from OZR compared to LZR (Figure 3B). Finally, to determine whether the increase in ASBT expression was at the level of the BBM as suggested by the kinetic studies above, ASBT was measured by Western blot in villus cell BBM and, as shown in Figure 3C, ASBT was increased in the BBM from OZR compared to LZR. Thus, the mechanism of stimulation of ASBT during obesity in Zucker rats is secondary to increased BBM cotransporter numbers. 

### 3.4. Regulation of ASBT in TALLYHO Mouse Model of Obesity

**Figure 4:**
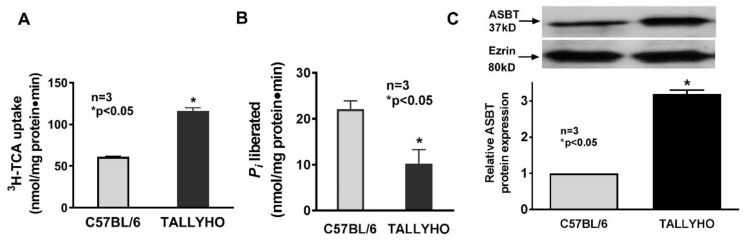
Alterations in Na–bile acid cotransport and Na-K-ATPase in villus cells from TALLYHO mice model of obesity. (**A**) Na–bile acid cotransport was significantly increased in villus cell BBM from TALLYHO mice compared to C57BL/6 mice. (**B**) Na-K-ATPase activity was significantly reduced in villus cell homogenates from TALLYHO compared to C57BL/6 mice. (**C**) ASBT protein expression was significantly increased in villus cell BBM from TALLYHO mice compared to C57BL/6 mice.

To determine if the observations in obese Zucker rats were also found in different species and models of obesity, the TALLYHO mice model of obesity was studied. Functional studies demonstrated that ASBT-mediated Na-taurocholate uptake was significantly stimulated in villus cell BBMV from TALLYHO mice compared to C57BL/6 (Figure 4A: 61 ± 0.8 nmol/mg pro/min in C57BL/6 and 116 ± 4 in TALLYHO; *n* = 3, *p* < 0.05). Also, as with Zucker rats, Na-K-ATPase activity was significantly reduced in the villus cell homogenates from TALLYHO mice compared to the controls (Figure 4B: 22.1 ± 1.8 nmol/mg pro/min in C57BL/6 and 10.2 ± 3.1 in TALLYHO; *n* = 3, *p* < 0.05). Thus, the mechanism of stimulation of ASBT in the TALLYHO mice model of obesity is not secondary to the altered Na-extruding capacity of the villus cells during obesity. Furthermore, comparable to Zucker rats, a significant increase in BBM ASBT protein expression (Figure 4C) was seen in TALLYHO mice. Therefore, the mechanism of stimulation of ASBT during obesity in TALLYHO mice also appears to be secondary to increased BBM cotransporter numbers. 

### 3.5. Expression of ASBT in Human Ileal Mucosa

**Figure 5:**
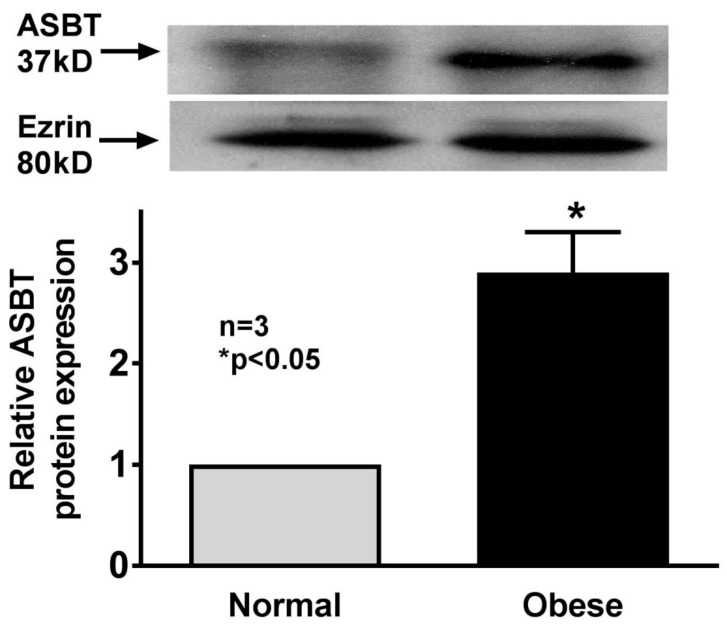
Na–bile acid cotransport expression in human distal ileal villus cells. ASBT expression was significantly increased in villus cells from obese human ileum compared to normal BMI human ileum.

Western blot experiments performed with human ileal mucosal protein extracts showed that ASBT protein expression was significantly increased in mucosal samples obtained from obese humans compared to nonobese humans (Figure 5).

### 3.6. Expression and Role of FXR in Obesity In Vivo

**Figure 6:**
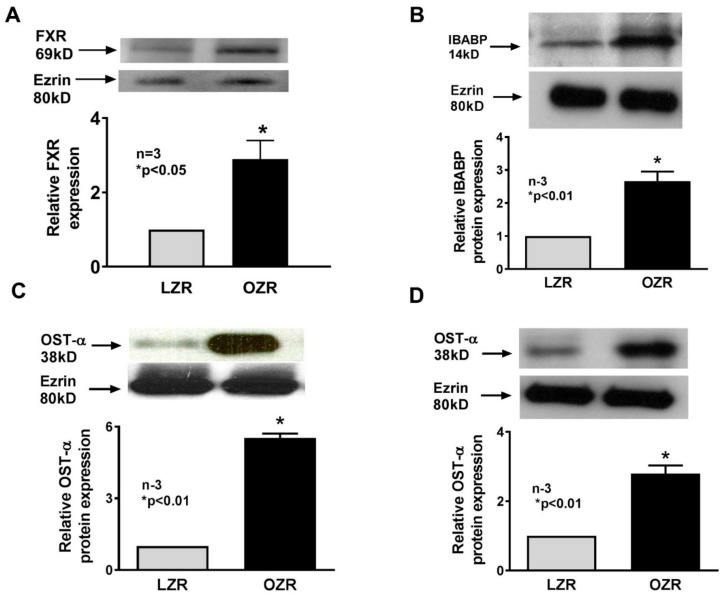
FXR and bile acid handling protein expression in Zucker rat villus cells. (**A**) FXR expression was significantly increased in ileal villus cells from OZR compared to LZR. (**B**). Ileal bile acid binding protein levels were also significantly increased in villus cells from OZR compared to leans. (**C**) Organic solute transporter alpha (OSTα) protein expression was significantly increased in villus cells from OZR compared to LZR. (**D**) OSTα protein expression was also increased in villus cell plasma membrane from OZR compared to LZR.

Since FXR is a known transcriptional regulator of ASBT, its levels were determined in villus cells. FXR levels were significantly increased in OZR compared to LZR (Figure 6A). FXR can also regulate the transcription of bile-acid-handling proteins to accommodate the result of ASBT stimulation in obesity. Thus, we looked at ileal bile-acid-binding protein (IBABP) in villus cells. IBABP levels were increased in villus cells in obese Zucker rats (Figure 6B). Bile acid export into the portal venous system from the villus cells occurs via solute transporter (OST). FXR is capable of stimulating the expression of OST. In obese Zucker rats, the levels of the alpha subunit of OST were increased in villus cells (Figure 6C). OSTα is a basolateral membrane protein and thus we looked at its expression in the plasma membrane, and indeed, OSTα was increased as well as in the plasma membrane (Figure 6D). These studies indicate that perhaps via the stimulation of FXR, ASBT, IBABP, and OSTα are transcriptionally enhanced during obesity.

### 3.7. Role of FXR in Obesity In Vitro

**Figure 7:**
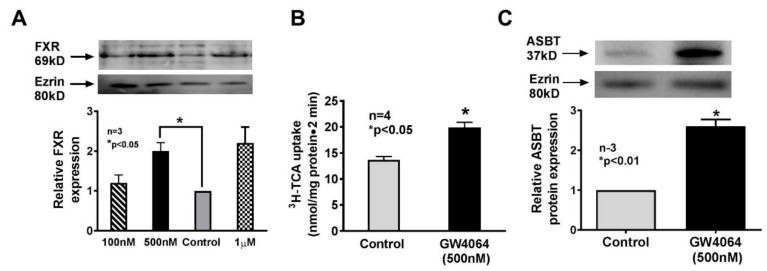
Effect of FXR agonist on rat intestinal epithelial cells (IEC-18) in vitro. (**A**) FXR agonist dose response effect on FXR expression in IEC-18 cells. 500 nM appeared to be the ideal dose of the agonist, which was used in all subsequent studies. (**B**) FXR agonist stimulated Na-dependent bile acid uptake in IEC-18 cells. (**C**) FXR agonist significantly increased IEC-18 cell BBM ASBT expression.

From the above in vivo studies it appeared that FXR has an important regulatory role in bile-acid-handling proteins. To ensure its significance, in vitro studies were carried out. Multiple doses of FXR agonist, GW4064, were used to determine the ideal dose of this agent to produce a significant increase in the expression of FXR in rat intestinal epithelial cell line IEC-18. It appeared that 500 nM GW4064 was the ideal dose, which was used in subsequent experiments (Figure 7A). FXR agonist significantly stimulated Na-dependent ^3^H-taurocholate uptake (Figure 7B) similar to that seen in OZR villus cells. Further, GW4064 significantly increased BBM ASBT expression in IEC-18 cells (Figure 7C). These data indicated that FXR most likely mediated the stimulation of ASBT in villus cells in OZR.

**Figure 8:**
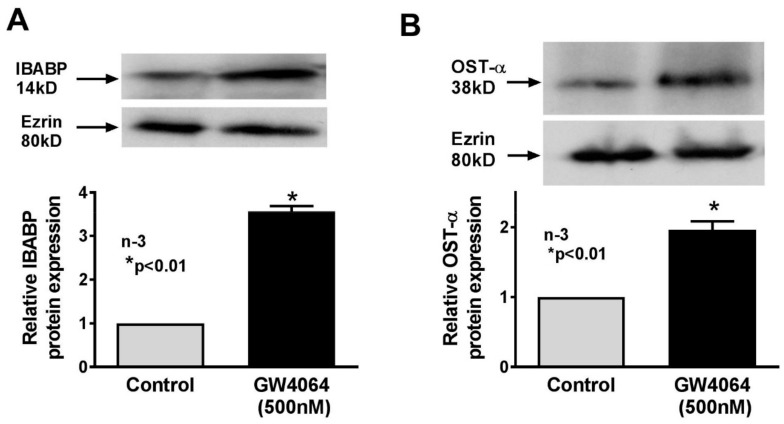
Effect of FXR agonist on bile-acid-handling protein expression in IEC-18 cells in vitro. (**A**) FXR agonist significantly increased IBABP expression in IEC-18 cells. (**B**) FXR agonist significantly increased OSTα expression in IEC-18 cells.

**Figure 9:**
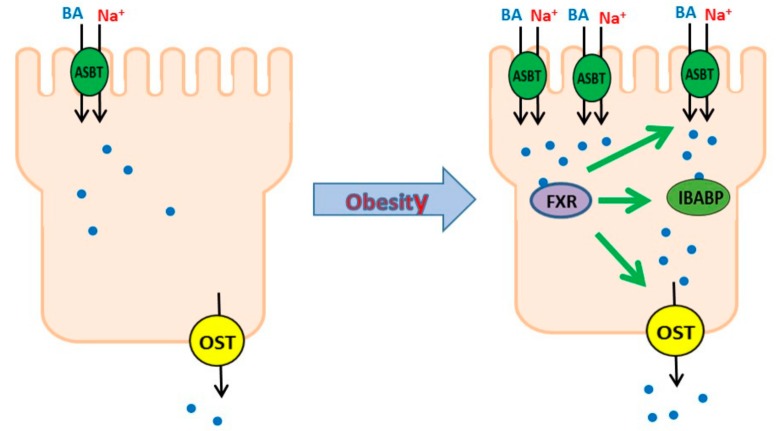
Effect of obesity on bile acid cotransport in intestinal villus cells. Obesity stimulates ASBT and other bile-acid-handling proteins likely via the stimulation of FXR in intestinal villus cells.

To determine if FXR also affected bile-acid-binding proteins during obesity comparable to what was observed in vivo, we studied IBABP and OSTα. FXR agonist significantly increased the cellular expression of IBABP in IEC-18 cells (Figure 8A). FXR agonist also significantly increased the cellular expression of OSTα in IEC-18 cells (Figure 8B). These data indicated that increased FXR expression likely mediated the increase in bile-acid-binding proteins IBABP and OSTα in villus cells during obesity. Thus, the increased expression of ASBT, as well as bile-acid-handling protein IBABP and bile acid export protein OSTα, all appear to be mediated by enhanced FXR expression during obesity (Figure 9).

## 4. Discussion

In the mammalian intestine, Na-dependent bile acid cotransport or ASBT expression is found in villus but not crypt cells [45,46]. Further, ASBT expression is restricted to the terminal ileum in mice, hamster, rats, and humans [46,47,48,49,50]. The importance of ASBT has been demonstrated in idiopathic diseases where mutations in ASBT have been identified as the cause of primary bile acid malabsorption. In these conditions, patients with primary bile acid malabsorption present with chronic diarrhea and steatorrhea from infancy resulting in fat-soluble vitamin malabsorption and reduced plasma lipid levels [9]. Much more common are conditions characterized by chronic intestinal inflammation such as inflammatory bowel disease (IBD or Crohn’s disease), where bile acid malabsorption is known to occur secondary to the inhibition of ASBT [12,51,52,53]. In IBD, cytokines are thought to decrease ASBT gene expression by acting through AP-1 (activator-protein-1), which binds a c-jun/c-fos heterodimer. Thus, during chronic intestinal inflammation, c-fos is phosphorylated and translocates into the nucleus where binding of the AP-1 element leads to transcriptional repression of ASBT expression [21,54]. Other conditions that involve ASBT-mediated intestinal bile acid malabsorption include hypertriglyceridemia, idiopathic chronic diarrhea, gallstone disease [10,55,56,57], post cholecystectomy diarrhea, and irritable bowel syndrome [58].

So while the importance of ASBT for bile acid absorption and the resulting lipid absorption/homeostasis is fairly evident, interestingly, very little is known about how ASBT may be affected in obesity where dyslipidemia is common and the consequential morbidities are numerous. Emerging evidence suggests altered bile acid assimilation as a potential factor in obesity-associated conditions such as type 2 diabetes mellitus and nonalcoholic fatty liver disease [59,60,61,62]. Further, increased dietary lipid absorption resulting in altered lipid homeostasis of obesity has been attributed to increased bile acid levels in enterohepatic circulation [5,6,7]. However, this study, for the first time, directly demonstrates how ASBT is affected in animal and human obesity that may result in the dyslipidemia of obesity.

In the Zucker rat model of obesity, ASBT activity was significantly increased in intact ileal villus cells. This increase in ASBT was not secondary to the altered Na-extruding capacity of the villus cells as Na-K-ATPase activity was not increased but was, in fact, decreased. Moreover, increased ASBT activity was present in the villus cell BBM from obese Zucker rats. At the level of the cotransporter in the BBM, kinetic studies demonstrated that the mechanism of increased taurocholate uptake was secondary to an increase in the number of cotransporters rather than an alteration of the affinity of the cotransporter. Western blot studies confirmed these findings by demonstrating an increased expression of ASBT protein in the villus cell BBM. Thus, the stimulation of ASBT during obesity may be secondary to enhanced de novo synthesis or trafficking of ASBT to BBM.

While Zucker obese rats are a useful model of obesity, they represent a rare form of monogenic obesity (leptin receptor gene deficiency). The common form of human obesity is generally accepted to be polygenic with environmental components. TALLYHO mice, a polygenic model of obesity, has been shown to develop moderate levels of obesity characterized by increased fat mass, insulin resistance, glucose intolerance, type 2 diabetes, hyperlipidemia, and hyperglycemia, which encompass many aspects of human obesity [32,33,34]. The ASBT findings in Zucker rats were further substantiated in the TALLYHO mice model of obesity. Here also a significant increase in ASBT activity and BBM ASBT expression was noted during obesity. Na-K-ATPase levels were also found to be decreased in villus cell homogenates from TALLYHO mice compared to C57BL/6 mice. Thus, in the TALLYHO mouse model of obesity, ASBT regulation was also comparable to that seen in the rat model of obesity. Finally, it is important to determine if the alterations seen in the animal models of obesity represent comparable changes in ASBT protein expression in human obesity. Indeed, ASBT protein expression was significantly increased in ileal villus cells from obese patients. Thus, ASBT appears to be stimulated in obesity secondary to increased expression in villus cell BBM.

Conflicting results have been reported regarding the regulation of ASBT expression by bile acids, including that ASBT is stimulated, inhibited, or unaffected by bile acids [63,64,65,66,67]. Bile acids have been shown to inhibit ASBT expression in mouse and rabbits acting through FXR and SHP to antagonize LRH-1, which is important for ASBT transcription [68]. The importance of LRH-1 is demonstrated by the observation that ASBT expression is diminished in the ileum of intestine-specific LRH-1 null mice [69]. Other studies have shown that activation of the nuclear receptor farnesoid X receptor (FXR) by bile acids enhances the transcription of ASBT [34]. In addition to FXR, transcription factors such as hepatocyte nuclear factor 1α (HNF-1α) and activator protein-1 (AP-1) have been shown to regulate ileal bile acid uptake through transcriptional induction of ASBT [21,22]. In human tissues, bile acids were found to act via the FXR-SHP pathway to antagonize RARα and decrease human ASBT transcription [70]. In contrast, bile acids have also been shown to increase ASBT transcription through an FXR-independent pathway involving the EGF receptor and mitogen-activated protein kinase/extracellular signal-regulated kinase signaling through an AP-1 element in the ASBT promoter [67]. Interestingly, unlike LHR-1, FXR does not appear to be necessary for ASBT’s basal expression; ASBT expression is not decreased in the ileum of FXR-null mice [71]. Differences between experimental designs such as the dose and mode of delivery of bile acid, methods for measuring ASBT protein or activity, species, and genetic background may account for some of the dissimilar findings to date [72,73]. In the present study, FXR was noted to be increased in villus cells from obese Zucker rats. To determine whether this increased FXR expression may have mediated the increase in ASBT during obesity, the effect of FXR agonist, GW4064, was studied in rat intestinal epithelial cells (IEC-18) in vitro. Based on the dose response study, 500 nM of the agonist reproducibly increased FXR expression in IEC-18 cells. This dose of the agonist also increased both Na-dependent taurocholate uptake in IEC-18 cells and the expression of ASBT in IEC-18 cell BBM. These data indicated that FXR is likely responsible for the enhanced transcriptional expression of ASBT in obesity.

Transcriptional regulation of ASBT has been well documented in other conditions. For example, exogenous corticosteroids can induce precocious expression of ASBT and also upregulate ASBT expression in mature animals [74]. But, interestingly, this regulation by glucocorticoids appears to be lost during chronic intestinal inflammation where corticosteroids have had a long-standing use in the treatment of inflammatory bowel disease. Coon et al. have demonstrated that while methyl prednisolone enhances the expression of ASBT in rabbits, in the rabbit model of IBD, glucocorticoids appear to reverse the inhibition of ASBT by likely inhibiting the immune inflammatory mediator responsible for the ASBT inhibition and not by directly enhancing the transcription of ASBT as was the case in normal rabbits [16]. In the streptozocin-induced rat model of diabetes mellitus, expression of ileal ASBT and other intestinal bile acid transporters was significantly elevated. More specifically, ASBT expression was increased by its increased promoter activity [24,25].

Since FXR is also known to affect other bile-acid-binding proteins, they were studied in vivo in Zucker rats and in vitro with the FXR agonist in IEC-18 cells. IBABP was increased in villus cells from obese Zucker rats in vivo and the FXR agonist increased IBABP in IEC-18 comparably in vitro as well. Similarly, OSTα in vivo was increased in villus cells from obese Zucker rats and the FXR agonist increased it in vitro in IEC-18 cells. These results indicate that during obesity, the increase in FXR stimulates not only the assimilation of bile acids by increasing ASBT, but also the subsequent intracellular handling and export of said bile acids by augmenting the appropriate bile-acid-handling proteins such as IBABP and OSTα.

The results of this study clearly demonstrate that at the cellular level, ASBT and other bile-acid-binding proteins are increased during obesity. However, what is yet to be determined is whether the caudal–oral distribution of ASBT is increased in obesity as well. Regulation of ASBT expression along the longitudinal axis of the intestine is not completely understood. Nevertheless, studies have demonstrated that transcription factor GATA4 is essential for this process. Indeed, intestine-specific inactivation of GATA4 in mice results in a dramatic induction of ASBT expression in proximal intestine [75,76]. Further, attenuation of gut microbiota has shown to alter the expression of ASBT by GATA4, increasing absorption of bile acids [23]. Thus, if GATA4 is critical for expression of ASBT along the cephalocaudal axis of the small intestine, this may be a novel mechanism for inducing proximal bile acid absorption by modulating the GATA4 pathway in bile acid malabsorptive conditions such as ileal resection.

In conclusion, in obesity, intestinal bile acid absorption is increased secondary to increased expression of apical bile acid cotransporter, ASBT, in the BBM of villus cells. The mechanism of stimulation of ASBT expression is likely secondary to increased expression of FXR in these cells. To accommodate the resultant increased assimilation of bile acids, FXR also increases bile-acid-binding protein IBABP expression as well as bile acid exit transporter OSTα in the BLM of these cells during obesity. Thus, this study demonstrated for the first time that in an epidemic condition, obesity, the dyslipidemia that leads to many of the complications of the condition, may, at least in part, be due to deregulation of intestinal bile acid absorption. 

## Figures and Tables

**Table 1:** Kinetic parameters of Na-taurocholate cotransport in the BBMV of villus cells from OZR and LZR. The maximal rate of uptake was found to be significantly increased in the villus brush border membrane vesicles from OZR compared to that from LZR (*n* = 4; * *p* < 0.05). However, the affinity for bile acid uptake remained unchanged between the two experimental conditions.

Kinetic parameters	LZR	OZR
*V_max_* (nmol/mg pro 15 s)	32.2 ± 0.9 *	63.5 ± 1.43 *
*K_m_* (µM)	71.3 ± 2	72.5 ± 5.9

## Abbreviations

ASBT	apical sodium bile acid cotransporter
LZR	lean Zucker rats
OZR	obese Zucker rats
BBM	brush border membrane
BBMV	brush border membrane vesicles
TCA	taurocholate/taurocholic acid
FXR	farnesoid X receptor
